# An Efficient Minimum Free Energy Structure-Based Search Method for Riboswitch Identification Based on Inverse RNA Folding

**DOI:** 10.1371/journal.pone.0134262

**Published:** 2015-07-31

**Authors:** Matan Drory Retwitzer, Ilona Kifer, Supratim Sengupta, Zohar Yakhini, Danny Barash

**Affiliations:** 1 Department of Computer Science, Ben-Gurion University, Beer-Sheva, 84105, Israel; 2 Agilent Laboratories, Tel Aviv, Israel; 3 Department of Physical Sciences, Indian Institute of Science Education and Research Kolkata, Mohanpur, 741246, India; 4 Microsoft R&D Center, Herzliya, Israel; 5 Laboratory of Computational Biology, Computer Science Department, Israel Institute of Technology, Haifa, 32000, Israel; Tel Aviv University, ISRAEL

## Abstract

Riboswitches are RNA genetic control elements that were originally discovered in bacteria and provide a unique mechanism of gene regulation. They work without the participation of proteins and are believed to represent ancient regulatory systems in the evolutionary timescale. One of the biggest challenges in riboswitch research is to find additional eukaryotic riboswitches since more than 20 riboswitch classes have been found in prokaryotes but only one class has been found in eukaryotes. Moreover, this single known class of eukaryotic riboswitch, namely the TPP riboswitch class, has been found in bacteria, archaea, fungi and plants but not in animals. The few examples of eukaryotic riboswitches were identified using sequence-based bioinformatics search methods such as a combination of BLAST and pattern matching techniques that incorporate base-pairing considerations. None of these approaches perform energy minimization structure predictions. There is a clear motivation to develop new bioinformatics methods, aside of the ongoing advances in covariance models, that will sample the sequence search space more flexibly using structural guidance while retaining the computational efficiency of sequence-based methods. We present a new energy minimization approach that transforms structure-based search into a sequence-based search, thereby enabling the utilization of well established sequence-based search utilities such as BLAST and FASTA. The transformation to sequence space is obtained by using an extended inverse RNA folding problem solver with sequence and structure constraints, available within RNAfbinv. Examples in applying the new method are presented for the purine and preQ1 riboswitches. The method is described in detail along with its findings in prokaryotes. Potential uses in finding novel eukaryotic riboswitches and optimizing pre-designed synthetic riboswitches based on ligand simulations are discussed. The method components are freely available for use.

## Introduction

### Overview

Genetic control of fundamental processes such as transcription, translation, and splicing is a complex process, and is in many cases mediated by proteins that monitor the environment and selectively bind to targets. Surprisingly, cis-acting RNA genetic control elements have been discovered that are capable of directly sensing small ligands thereby playing a regulatory role by switching conformational states without the participation of proteins. These RNA elements are called riboswitches. Although the first experimental validations were published in 2002 [1–4], conserved sequence patterns in the 5' UTRs of bacteria were identified several years earlier using comparative analysis of the upstream regions of several genes expected to be co-regulated. These studies contributed to the description of the RFN element [5], the S-box [6] and the THI-box [7].

One of the first riboswitches to be discovered, the ‘TPP-riboswitch’, provides an RNA control mechanism for both transcription termination and translation initiation during thiamine (vitamin *B*_1_) biosynthesis in bacteria. It is an RNA element that responds to concentration changes of thiamin pyrophosphate (TPP) with a conformational rearrangement that affects transcription termination in *Bacillus subtilis* and translation initiation in *Escherichia coli*. Other discovered riboswitches respond to molecules that change conformation upon binding like flavin mononucleotide (FMN), S-adenosylmethionine (SAM) [8,9], coenzyme B_12_, lysine, guanine, adenine, and later some more peculiar riboswitches including glmS, glutamine, glycine, cyclic di-GMP [10,11]. The structural basis and biochemical properties of several of these riboswitches have been elucidated at high resolution (for example see [12–14] for the glycine riboswich, [15,16] for the glmS riboswitch, and [17–21] for the cyclic di-GMP riboswitch). All the above riboswitches were discovered solely in prokaryotic organisms. The ‘TPP-riboswitch’ is the only riboswitch class found in plants and fungi, yet no evidence indicates its existence in higher eukaryotes. The identification of a wider spectrum of riboswitches with a more significant representation among eukaryotes relative to what is known at present [22,10] remains a challenging task. To achieve this goal, there is interest in developing improved computational search methods for riboswitch discovery. From the bioinformatics standpoint, a substantial amount of information can be inferred about riboswitch mechanism by examining its structure as well as its sequence. The conserved sequence and structure of the aptamer domain can identify riboswitches with analogy to a fingerprint, a fact that can be utilized by structure-based bioinformatics search methods that also involve sequence considerations.

Some bioinformatics contributions in the context of riboswitch search methods are noteworthy. A simple search program called SequenceSniffer was used by Barrick and Breaker early on to identify new riboswitches in bacteria as well as most of the few examples of eukaryotic riboswitches known to date [23]. Subsequent work on a genomic scale by Barrick et al. [24,25] triggered many more findings of bacterial riboswitches. Weinberg and Ruzzo helped discover new classes of riboswitches, through a covariance models (CM) approach implemented in CMfinder [26,27], applied biologically in [28–32]. The insertion of known riboswitches into the Rfam database [33–35] was also instrumental in advancing the field. Gelfand and coworkers [36] have continued advancing the comparative analysis approach in microbial communities, with more recent findings using comparative genomics in metagenomes [37,29,32]. Other simpler sequence-based methods have also been developed [38,39], the more sophisticated of them employs sequence-based filters for detecting new riboswitches [40]. Another approach is that of RSEARCH [41], which by stochastic context-free grammar, can also be used to search for new riboswitches. Hidden Markov Models (HMMs) are frequently used to search for new riboswitches such as in pHMM [42] and the advanced Infernal, which also incorporates covariance models [43–45]. Other approaches include Boltzmann probability of RNA structural neighbors for riboswitch detection as in RNAbor [46]. Genome-wide measurement of RNA secondary structure by high-throughput sequencing [47] can also be useful in attempting riboswitch discovery. On the topic of searching for new riboswitches in genomes, a review is available [48]. There are also new ways being developed to detect riboswitches using their 3D structural modules [49].

Here, we present a novel structure-based riboswitch detection method that incorporates energy minimization techniques for riboswitch searching [50] while also taking into account conservation in sequence. Our approach, developed independently, follows the same philosophy as that of [51] where a method to identify IRES-like structural domains without considering riboswitches was put forth. For this, we use folding prediction algorithms such as *mfold* / *UNAFold* [52–54] or the Vienna RNA package [55–57] with the most recent energy rules [58]. Reference [50] provides a preliminary focused review about using folding prediction methods in the context of riboswitches. Energy minimization was used to identify a potential purine riboswitch in *Arabidopsis thaliana* that exhibited some basic properties shown in [59–61] and was tested biochemically using in-line probing [62]. We also note that another similar case where an attractive candidate was suspected to be a SAM riboswitch in vertebrates was reported in [63] using comparative analysis and was tested experimentally because of its interest. From the computational perspective, the main drawbacks of existing structure-based methods are the repeated use of folding predictions within a moving window with a fixed window size throughout a sizeable data set. This approach leads to high computational time complexity and to limited accuracy of the folding predictions performed on the data set (only the accuracy of the folding prediction performed on the query can be checked in advance). These drawbacks are remedied by the method we propose and describe herein.

### Preview on traditional search methods for riboswitches

The traditional methods that are mostly sequence-based are still useful when analyzing a newly sequenced genome (e.g., as in [64]). These include methods like SequenceSniffer that was used earlier on for the detection of more prokaryotic riboswitches [65,24] and the initial detection of eukaryotic riboswitches [23], as well as the state-of-the art Infernal that supports riboswitch searches using Covariance Models and Hidden Markov Models [44,45]. Development of efficient new structure-based methods is important for advancing further, also for *de novo* discovery of structured ncRNA in general [66]. Here, we focus on RNA secondary structure when referring to structure-based methods, but this can also expand to RNA tertiary structures as in RMDetect, developed by Cruz and Westhof [49].

### Motivation for energy-minimization structure-based search methods for riboswitches

While some of the aforementioned search methods for riboswitches contain a certain amount of structural consideration, we believe that in order to find additional examples of eukaryotic riboswitches it is important to put a considerable emphasis on structure (without neglecting sequence conservation). This is because searching for a known consensus query pattern from bacteria in a eukaryotic database with sequence-based methods is from the evolutionary standpoint expected to be less fruitful than searching the same prokaryotic consensus in a database of prokaryotic organism. Structure conservation should play a more dominant role in the delicate balance between sequence and structure when patterns from more distant organisms are aligned. A well-known structure identifier program called RNAMotif [67] is available but it is a descriptor language that cannot be easily modified for the purpose of riboswitch searches.

Motivated by addressing this challenge, a method has been developed for eukaryotic riboswitch discovery that is highly structure based [50,68,62,69]. This method utilizes consecutive energy minimization predictions with a sliding window, that each time compares the predicted structure of the query with the predicted structure of the sequence data inside the window segment. Because energy minimization predictions are computationally expensive, the method cannot be used genome wide. Another deficiency in this approach is that while folding prediction of the riboswitch aptamer (being the query sequence) can be checked by comparing its structure to a biological experiment, the folding prediction within each window segment when scanning the sequenced data cannot be checked for its accuracy. This limits its use to only special cases of small sequenced data, such as when focusing on a certain metabolic pathway and genes that are associated with it. In such a case, the method can be employed with high resolution to look only in genes that play a role in that specific metabolic cycle (e.g., in purine metabolism, as demonstrated in [50]).

## Methods

### A new energy minimization structure-based search method for riboswitches

The method described in this paper was devised to remedy shortcomings in the efficiency of existing energy-minimization structure-based search methods discussed above, also outlined and exemplified in [50]. Importantly, while all other methods scan the target sequence data using a moving window, repeatedly performing expensive energy minimization predictions, this step is completely circumvented in our method, allowing for an extensive genome wide scan rather than being restricted to a single gene or a pathway (e.g., purine metabolism as in [50]).

Our new approach relies on the fact that the aptamer domain of riboswitches is well-conserved in evolution in both sequence and structure. Sequence-based methods such as BLAST should thus be an integral part of riboswitch detection as they are easily available, highly efficient, and can partially address riboswitch conservation. However, sequence-based methods alone or with slight structure considerations have been exhausted for this task. Thus, we start from a structure-based approach and use our in-house inverse RNA folding program RNAfbinv [70] (available at http://www.cs.bgu.ac.il/~RNAexinv/RNAfbinv) to transform the search task into sequence space. RNAfbinv receives as input a secondary structure along with sequence constraints, and outputs a set of designed sequences that are predicted to be consistent with the input structure. This set of sequences can then be searched using BLAST. A similar idea was used in [51] to identify IRES-like structural domains using the inverse folding algorithm RNAiFOLD [71]. The flexibility of RNAfbinv [70] opens new possibilities in sampling the search space that are not covered by any other method developed before because a few more or less nucleotides in certain fragments are allowed in the solution. A summary of the computational method for the RNA inverse folding solver we used and the ingredients for RNAfbinv are provided in the next sub-section.

**Fig 1:**
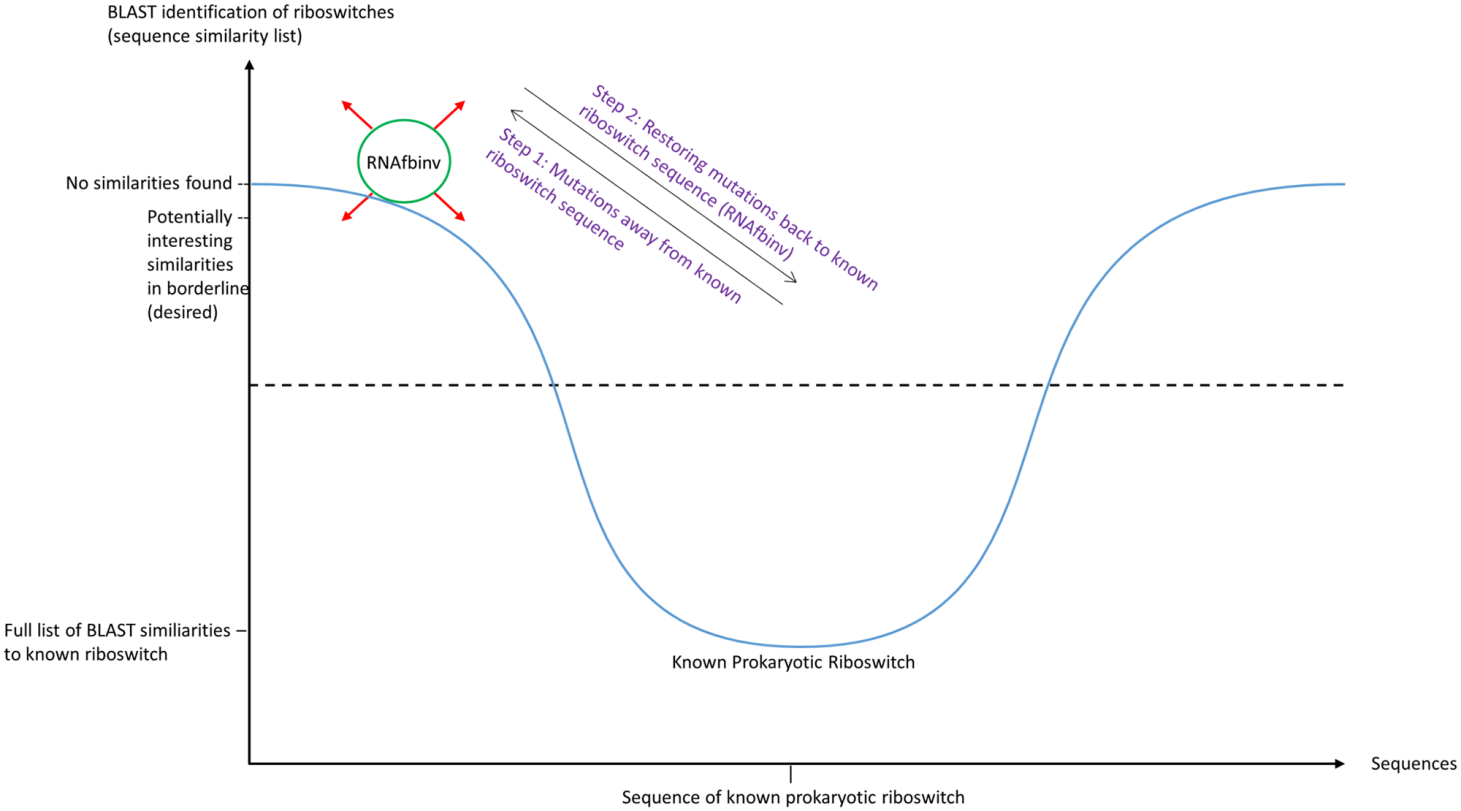
Schematic illustration of a potential well with a minimum in the known aptamer sequence. It is possible to escape the minimum by performing nucleotide mutations to the initial sequence until there are no BLAST hits when inserting the mutated sequence as input. If the mutations disrupted the known aptamer structure, the use of RNAfbinv will restore the known structure while generating designed sequences as output. Subsequently, designed sequences in the borderline of the potential well that do show BLAST hits should then be carefully examined in their hits. It is expected that most of the hits observed will be from known bacteria but a few unknown bacteria and exceptional eukaryotic organisms will also show up.

**Fig 2:**
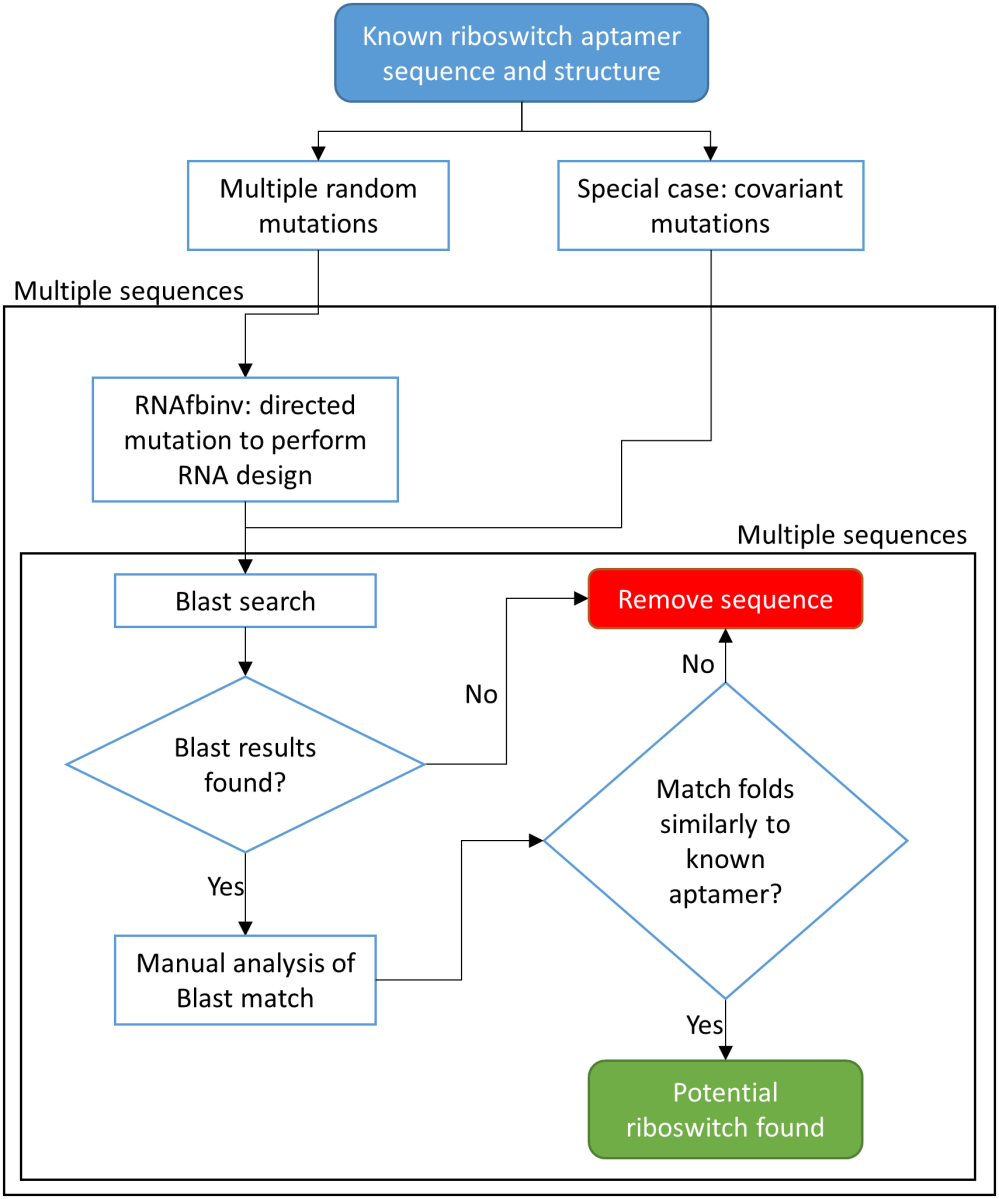
Method illustration. Starting from a known aptamer sequence, performing random nucleotide mutations to escape from the similarity of the known aptamer sequence, some of the mutations may disrupt the aptamer structure (See Fig 1), followed by directed mutations that restore its structure using RNAfbinv [70] and offer newly designed sequences for inspection. During these phases we generate multiple designed sequences. The designed sequences are BLASTed against large DNA databases such as NCBI's Nucleotide colletion nr/nt. Similar BLAST results are folded using the Vienna RNA package [57] and compared visually to the known aptamer secondary structure. Similar sequences are marked as potential aptamers. The special case of covariant mutations explores sequences that fold to the same structure, thereby not allowing for the flexibility of the fragment-based design.

The initial step of the pipeline presented herein is to start from the sequence of an experimentally verified aptamer domain and perform the following two tests: (1) RNA folding predictions by energy minimization (e.g., with mfold or RNAfold from the Vienna RNA package) should match the experimental result to a fair extent (user estimation); (2) BLAST search on the initial riboswitch sequence produces known locations in various genomes that are familiar for the known aptamer at hand. In the next stage, random nucleotide mutations (e.g., changing 'A' in a certain position to either 'C', 'G', or 'U') are introduced successively followed by a BLAST search on the mutated sequence. This step is performed iteratively until a sequence with no BLAST hits is reached. For this step we chose to use the option "Somewhat similar sequences (blastn)" that is available in the standard nucleotide BLAST utility at NCBI. At this point, the predicted structure of the wild-type aptamer is given as input to RNAfbinv along with desired constraints (importantly, the mutated sequence, and optionally a specific fragment that is essential to preserve such as the multi-branch loop of the purine riboswitch) in order to restore back the structure of the initial aptamer domain. The output of RNAfbinv now consists of designed sequences that are tested again using BLAST. The majority of the designed sequences are not expected to yield novel findings, either because they reached too far away or too close to the known aptamer in sequence space, but a few of the designed sequences that reached the borderline between no BLAST hits and some familiar BLAST hits (see illustration in Fig 1) may contain similarities to peculiar organisms in various genomes that are not typical to the riboswitch aptamer at hand (special bacteria, eukaryotes). Each of these similarities is then examined for its GenBank location in the genome, adding segments upstream and downstream from its location that match the size of the known initial aptamer, and comparing the folding prediction by energy minimization to that of the known aptamer. If the comparison is satisfactory up to a small structural difference of one or two base pairs, a riboswitch candidate is recorded. The above procedure can be semi-automated to facilitate an extended search in sequence space as depicted in the flow chart of Fig 2. The special case included on the right hand side of Fig 2 is also explained below.

To elaborate, the general procedure described in Fig 2 includes the following steps:

1. Perform successive single-point random nucleotide mutations (e.g., changing 'A' in a certain position to either 'C', 'G', or 'U') until there are no BLAST hits for the mutated sequence. Repeating several times, we obtain multiple random mutated sequences, before entering the outer black-framed box in Fig 2 containing the RNAfbinv step (neglecting for the time being the special case outlined on the right hand side of Fig 2 that is dealt with below).
2. Insert the predicted secondary structure of the initial aptamer along with each of the 500 mutated sequences as a starting point to RNAfbinv (instead of a random initial guess), each of the mutated sequences is sent to RNAfbinv 20 times. Independently, nucleotide constraints of essential nucleotides for ligand-binding are also inserted as input. The output of RNAfbinv are designed sequences for which the structure of the initial aptamer was restored, i.e., their predicted structure is similar to that of the initial aptamer. These designed multiple sequences appear before entering the inner black-framed box in Fig 2.
3. Perform a BLAST search on each of the designed sequences to look for new riboswitch candidates. Most of the BLAST results are expected to be known riboswitches in Rfam, but several that are not may be potential riboswitch candidates.

A special case of the above procedure can be performed using chosen covariant mutations (compensatory mutations performed on a base-pair, e.g. A-U to C-G, which are shape-preserving in line with the RNAfbinv approach). If an RNA inverse folding solver other than RNAfbinv is used that strictly preserves the secondary structure, both random nucleotide mutations that are performed in order to escape the potential well in Fig 1 and the directed nucleotide mutations that are performed by the RNA inverse folding solver in order to restore the initial aptamer structure should be made structure-preserving. In such a case, there is no distinction between random mutations to reach outside the well and directed mutations for structure restoration to get back inside, since all of the mutations are covariant mutations that preserve structure. This special case which only covers a small portion of the sequence search space is depicted on the right hand side of Fig 2. It is fast but it is not capable of reaching the vast majority of designed sequence results that can be achieved. Its main significance is that it can be used for illustrative purposes when introducing the method to practitioners without their need to download RNAfbinv.

The special-case procedure that is added as an option on the right hand side of Fig 2 includes the following steps:

1. Perform successive covariant mutations (compensatory mutations that are structure-preserving, e.g. changing 'A-U' to 'C-G', or single-point mutations in bulges or loops that do not alter the structure) until there are no BLAST hits for the mutated sequence. Repeating several times, we obtain multiple mutated sequences.
2. Perform reverse steps using covariant mutations. Note that this is a special-case to using RNAfbinv because an inverse RNA folding in the same orientation of RNAfbinv (shape-preserving and chosen fragment strict secondary structure-preserving) is performed on each of the mutated sequences, but since the mutated sequences are carefully selected to perform the same as RNAfbinv there is no need for RNAfbinv to restore the shape and chosen motif's secondary structure. Similarly to the RNAfbinv step in the general algorithm, the outputs of these mutation steps are designed sequences, with a predicted structure resembling that of the initial aptamer. These designed multiple sequences appear before entering the inner black-framed box in Fig 2.
3. Perform a BLAST search on each of the designed sequences to look for new riboswitch candidates. Most of the BLAST results are expected to be known riboswitches in Rfam, but several that are not may be potential riboswitch candidates.

### Method validation

There are thousands of known purine riboswitches currently available in Rfam, along with several comprehensive studies describing their phylogenetics and distribution in prokaryotes [72]. Aside from a small subset, neither these thousands of known purines nor new ones updated in Rfam have been validated experimentally. However, well-established software such as Infernal or pHMM has shown the ability to detect new purine riboswitch candidates with high score. Thus, when combined with convincing evolutionary justification, it is possible to conclude that such candidates are most probably true positives. In the following section we validate our method by presenting interesting result candidates that are not yet available in Rfam, are undetectable using BLAST or other sequence-based search methods alone, yet are detectable by a focused use of the pHMM model [42] applied to the newly whole-genome sequenced data of the candidates organisms. In addition, we provide convincing phylogenetic justification of their evolutionary similarity to known purine riboswitches [72].

### Computational method of the flexible RNA inverse folding solver used (RNAfbinv)

The inverse RNA folding problem for designing sequences that fold into a given RNA secondary structure was introduced in [55]. The approach to solve it by stochastic optimization relies on the solution of the direct problem [52,55]. Initially, a seed sequence is chosen, after which a local search strategy was used in the original RNAinverse of [55] to mutate the seed and repeatedly perform RNA folding prediction by energy minimization. Recently, aside of many important contributions by several groups in the field of RNA inverse folding (we refer the interested reader to [70] for a more detailed background), we developed an extension to the approach that allows designing sequences that fold into a prescribed shape, leaving some flexibility in the secondary structure of RNA motifs that do not necessarily possess a known functional role. The shape of the RNA is represented as a tree-graph [73] in our implementation. The RNAfbinv program that implements this type of sequence design, as described in [70], relies on programs from the Vienna RNA package such as RNAfold, RNAinverse, RNAdistance [55]. Basically, it starts with some initial sequence and minimizes the following objective function evaluated for candidate sequences compared to the target—the original riboswitch aptamer sequence and structure:

$$\begin{array}{l} \text{f(candidate, target)}=\\ \left|{\mathrm{neutrality}}_{\mathrm{target}}-{\mathrm{neutrality}}_{\mathrm{candidate}}|*100+|{\mathrm{dG}}_{\mathrm{target}}-{\mathrm{dG}}_{\mathrm{candidate}}\right|\\ +\mathrm{target}\_\mathrm{motif}\_\mathrm{exists(candidate)}*1000\\ +\mathrm{tree}\_\mathrm{edit}\_\mathrm{distance}\_\mathrm{shapiro}\_\mathrm{representation(target, candidate)}*100\\ +\mathrm{base}\_\mathrm{pair}\_\mathrm{distance}\_\mathrm{dotBracket}\_\mathrm{representation(target, candidate)}*0.01 \end{array}$$

The weights are fixed and the rationale for their values is explained below, as well as a description for each one of the terms. To start with, the extra term for the target motif existence is the most important constraint in general that should be fulfilled exactly without any compromise. Therefore a much larger weight of 1000 relative to all others is chosen for this term [70]. In our problem of riboswitch identification, we may use it in case we encounter a specific motif such as the multi-branched loop of the guanine-binding aptamer that we would like to preserve. The neutrality for measuring mutational robustness is a number between 0 and 1, therefore a weight of 100 is assigned. The minimum free energy dG is for measuring thermodynamic stability in kcal/mol, therefore a unity weight is assigned. Both these terms were not used in our current work because new riboswitches with different nucleotide compositions relative to the initial wild-type sequence may have different neutrality and thermodynamic stability. The distances between secondary structures are calculated using RNAdistance in the Vienna RNA package [55] (supporting both the coarse-grain tree graphs called the Shapiro representation [73], and the dot bracket representation of the secondary structure). For the tree edit distance between Shapiro representations, a relatively large weight of 100 is chosen for shape preservation, while for the base pair distance in the last term, a very small weight of 0.01 is assigned. This last term is the one used in the original RNAinverse [55] for preserving the exact secondary structure and its purpose is to protect the solutions from being over-dominated by shape. As explained in the Results and Discussion section in relation to Table 1, shape preservation that is controlled by the term with the weight of 100 (minimizing distances between shapes) is an important aspect of our method, allowed with a flexible RNA inverse folding solver. RNAfbinv uses simulated annealing with a four-nucleotide look ahead local search function.

**Table 1:** Results obtained with the purine riboswitch: new bacterial aptamer domains detected by our method.

Organism	Location in genome	Sequence similarity^a^ to *xpt*	Base pair distance (bp’s) to *xpt*	Shapiro distance to *xpt*
*Halobacillus halophilus* DSM 2266	596146–596213	Expect 8e-14;Identity 52/64(81%)	14	2
*Amphibacillus xylanus* NBRC 15112	401191–401257	Expect 2e-15;Identity 57/69(83%)	38	2
*Desulfosporosinus orientis* DSM 765	822336–822401	Expect 2e-16; Identity 56/65(86%)	35	2
*Pelosinus sp*.UFO1	1401846–1401911	No match	39	2

^a^ Sequence similarity information is taken from BLAST comparison.

## Results and Discussion

### Validation on bacteria with the purine riboswitch aptamer

For validating our proposed method, we chose to work with the purine riboswitch aptamer. This specific aptamer was found to be convenient to work with in the context of our method because its fold prediction by energy minimization is nearly identical to the experimentally derived structure [59–61]. Specifically, we use the *Bacillus subtilis xpt* guanine-binding aptamer domain. Thus, our initial aptamer sequence, of length 69 nts, is:

CACUCAUAUAAUCGCGUGGAUAUGGCACGCAAGUUUCUACCGGGCACCGUAAAUGUCCGACUAUGGGUG

**Fig 3:**
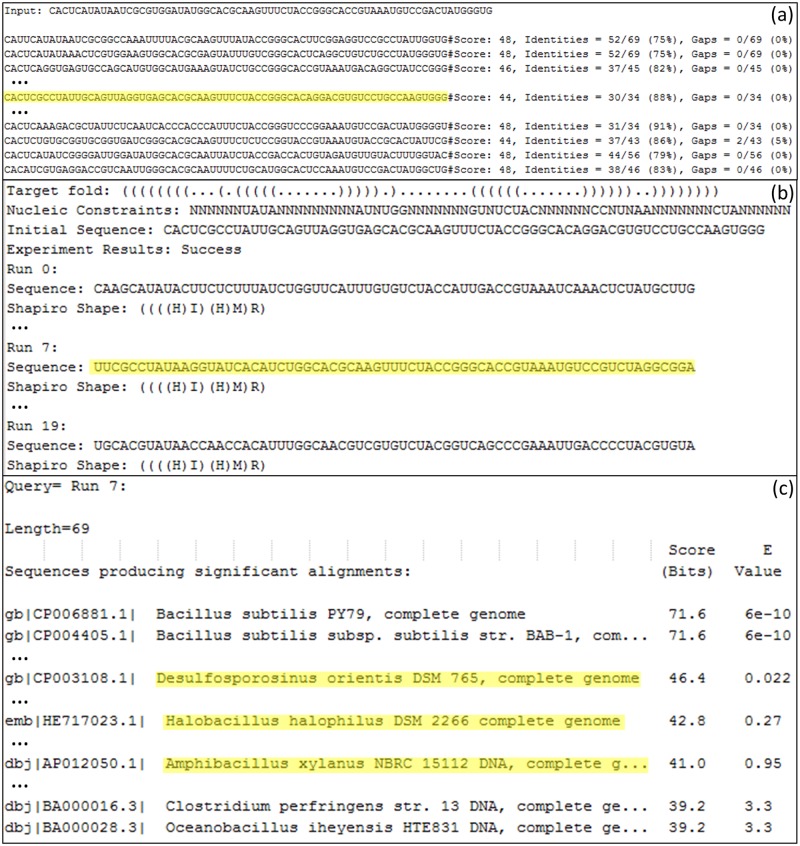
Outputs of the search flow shown in Fig 2. Selected outputs taken from the search, resulting in Fig 4. (a) Output screen for the multiple random mutation phase. On top, the initial known aptamer sequence–the *xpt* guanine-binding riboswitch aptamer, below are some of the generated sequences. (b) Output of multiple RNAfbinv runs. On top, the input including the starting sequence (marked in the frame above), below are designed sequences from multiple runs. (c) Output of nucleotide BLAST for input sequence of Run 7 (marked in the above frame), below are the BLAST matches including the three marked results that appear in Fig 4.

Using our general procedure, with RNAfbinv, as illustrated in Fig 2 and outlined in the previous sub-section, we obtain three peculiar bacterial candidates as depicted in Fig 3: *Desulfosporosinus orientis*, *Halobacillus halophilus and Amphibacillus xylanus*. Because RNAfbinv uses stochastic optimization, replicating these results may take time by the user, therefore for simplicity we continue discussing these findings using the special case of covariant mutations as depicted in the right hand side of Fig 2. This will allow the reader to replicate our three bacterial candidate results (Fig 3), which we obtained by using the general RNAfbinv procedure, without the actual need to run RNAfbinv to reach these specific results.

Therefore, for illustration purposes only, the special case of covariant mutations as depicted in the right hand side of Fig 2 is used. Covariation was also used in [74] for studying the purine riboswitch, but with the terminator and anti-terminator stems included to analyze alternative structure, whereas here we only consider the aptamer domain. Gradually mutating using only covariant mutations to get outside the potential well until we do not have any BLAST hits (the performed mutations are underlined) yields:

UGAAUGUAUAAUUCGACCUGGCAACGGUCGAAGUUUCUACCAAAAAAUAACCGUUUUUGACUACAUUCA

It should be noted that the covariant mutations are specifically selected to correspond to the RNAfbinv approach, which does not require to strictly preserve the secondary structure, but requires to preserve the shape and to strictly preserve the secondary structure of the chosen fragment. In the case of the purine riboswitch aptamer, for biological considerations we have chosen the constrained fragment to be the multi-branch loop where the ligand-binding occurs.

Next, mutating to get back to the borderline where we find BLAST hits for the first time, by restoring the right segment (the AAAA…UUUUs) towards the initial aptamer sequence, produces:

UGAAUGUAUAAUUCGACCUGGCAACGGUCGAAGUUUCUACCGGGCACCGUAAAUGUCCGACUACAUUCA

We then perform a BLAST search with the option "Somewhat similar sequences (blastn)" that is available in the standard nucleotide BLAST utility at NCBI on the above sequence, after exchanging to DNA letters:

TGAATGTATAATTCGACCTGGCAACGGTCGAAGTTTCTACCGGGCACCGTAAATG

TCCGACTACATTCA

The corresponding BLAST hits obtained contain more remote similarities than that of the initial starting sequence. The most interesting hit at first sight is that of *Drosophila melanogaster*, the only eukaryotic organism available in the bacteria-dominated list of hits. This hit appears among some distant bacillus types at the bottom of the hit list. Closer inspection of the surrounding of this hit in GenBank, adding more nucleotides upstream and downstream and then performing fold prediction by energy minimization on a similar-length, suitably aligned sequence reveals that it is a false positive hit. A similar case for the preQ1 riboswitch accompanied by a general discussion on the importance of structure to aptamer function is given in the next sub-section. All other hits from bacteria are true positives, almost all of which can be found in the Rfam database [34] as is available in the year 2014, except for *Desulfosporosinus orientis*, *Halobacillus halophilus and Amphibacillus xylanus*. These three bacteria were found by the above procedure using BLAST on a calculated pre-designed RNA sequence that is away from the initial aptamer sequence.

**Fig 4:**
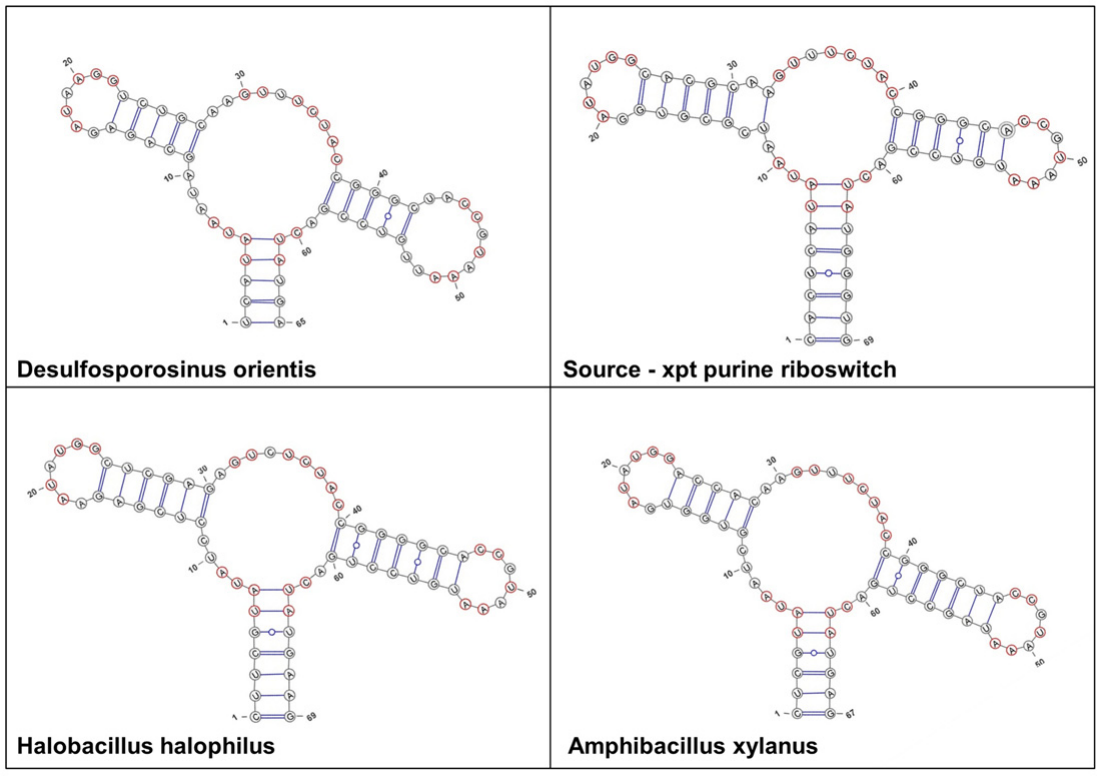
Results using the purine riboswitch for method validation. The predicted secondary structure drawings of three new bacterial aptamer domains detected by our method, in comparison to the known *xpt* guanine-binding aptamer domain. Shown in red are preserved nucleotides.

Further examination of the three predicted purine bacterial riboswitches, listed in Table 1, shows that they all belong to genomes whose sequences became available only recently in 2013, hence they are not yet available in Rfam at the time of our manuscript submission. In order to validate these findings, we used pHMM [42–72] and found that it is also able to detect the purine riboswitch upstream to the *xpt* gene in all the above three species with a high positive score. This result further substantiates our new method. The genome location of the three purine riboswitch bacterial candidates detected by our method as well as their quantitative similarity measures to the initial *Bacillus subtilis xpt* guanine-binding riboswitch domain are reported in Table 1. By inspecting their evolutionary origin and classification [72], one from the Clostridia class and the two others from the Bacilli class of the Firmicutes phylum, and closely examining their sequence and structure composition, it is expected that all three of them are indeed purine riboswitch guanine-binding aptamer domains. We note that Table 1 indicates large base pair distances between our bacterial findings and the original *xpt* guanine-binding riboswitch domain. This is caused by minor shifts in the connections between coarse-grained secondary structure motifs, emphasizing the importance of the Shapiro distance measure [73] which considers only the coarse-grained motif structure and accounts for the structural distance that is being minimized in the RNAfbinv step of our approach [70]. Fig 4 depicts their predicted secondary structures in comparison to the initial *Bacillus subtilis xpt* guanine-binding purine riboswitch domain. The multi-branch loop that is centered at the guanine-binding domain and supports the rest of the structure is up to four bases larger in all our findings. Our flexible method allows for slight changes (one or two base pairs) in the amount of base pairs in any motif, which is one of the advantages that can assist us in detecting new riboswitch candidates not yet found.

**Fig 5:**
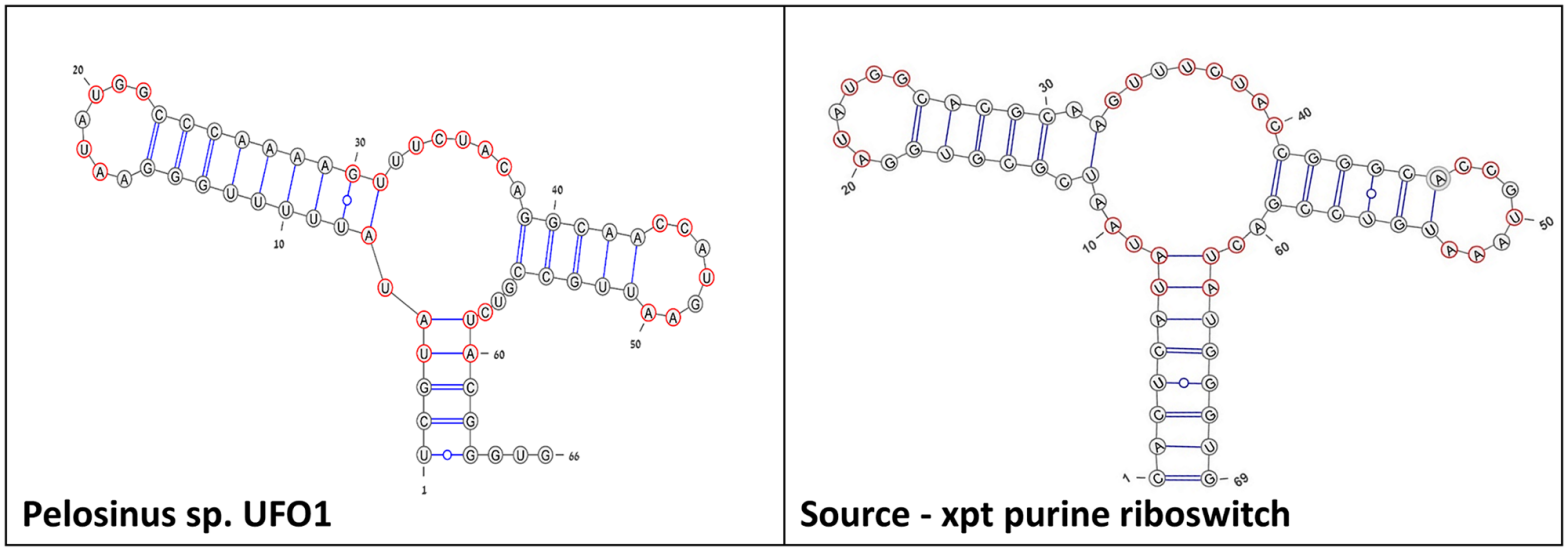
A potential prokaryotic new finding. The predicted secondary structure drawing of an additional new bacterial aptamer domain detected by our method, in comparison to the known *xpt* guanine-binding aptamer domain. The aptamer sequence does not align via BLAST with the *xpt* riboswitch. Shown in red are preserved nucleotides.

As mentioned above, the three candidates whose secondary structure is drawn in Fig 4 can be reached by the special case of covariant mutations without the need for RNAfbinv, but there are other candidates found only using the general procedure that includes RNAfbinv because the special case covers only a small amount of sequence space. One such bacterial candidate is the *Pelosinus* sp. Strain UFO1 that is compared to the *Bacillus subtilis xpt* guanine-binding purine riboswitch domain in Fig 5. We were not able to reach this candidate using the special case of covariant mutations. As viewed in Table 1, this bacterial candidate is more peculiar compared to the previous three because its aptamer sequence does not align via BLAST with the *xpt* riboswitch, as there is no sequence similarity between this candidate and the *xpt* riboswitch aptamer. It was found by a BLAST search on one of the designed sequences obtained as output from RNAfbinv. The candidate belongs to the Firmicutes phylum and from the evolutionary perspective, it does make sense that it is indeed a purine riboswitch [72]. Furthermore, we checked that this riboswitch candidate can also be detected using the pHMM model [42], with a fairly high pHMM score and a low E-value, but with the following peculiarity in comparison to the previous three bacterial candidates. A BLAST search was performed against the seed sequences used to build the pHMM model to check whether it is possible to get the new candidate sequence to align with any existing purine riboswitch sequence. It was possible to get it to align with only one out of 133 sequences present in the set, specifically a purine riboswitch belonging to the organism *Geobacillus kaustophilus* HTA426. This confirmed that the candidate sequence we found, illustrated in Fig 5, is indeed unusual and indicated that more simulations with our general procedure using RNAfbinv may yield a candidate that cannot be detected by computational riboswitch detection methods available to-date because they are less structure focused. We will present such a finding in the final sub-section of the current Results and Discussion section.

### The importance of structure consideration illustrated with the preQ1 riboswitch

Starting with the wild-type sequence of the *queC* riboswitch aptamer from *Bacillus subtilis* [75], which recognizes preQ1 and thus belongs to the general class of preQ1 riboswitches, structure-preserving (covariant) mutations were performed to migrate away from the wild-type in sequence space. The initial wild-type sequence as depicted in Fig 2D of [75] and transformed to DNA letters is:

CCGTGCGATATGCGGGAGAGGTTCTAGCTACACCCTCTATAAAAAACTAAGGA

Using the special case described in the right hand side of Fig 2, we perform gradual structure-preserving mutations to get outside the potential well. In one case we performed 17 single-point mutations and we still obtained BLAST hits for the preQ1 riboswitch using the following sequence (Hamming distance of 17 from the wild-type):

ACGTGCGATATGCGTGGGCCGTTCTAGCTACACCGGCCATTTTTTTCATTGGA

The same structure of the wild-type is retained, as indicated by *mfold* and *RNAfold* [53,56] on this sequence. However, when performing two additional single-point mutations in the form of a covariant mutation with the purpose of preserving the structure (replacing the base-pair C-G by A-T in the stem that is closest to the 5'-end) we get:

ATGTGCGATATGCATGGGCCGTTCTAGCTACACCGGCCATTTTTTTCATTGGA

When inserting this sequence to BLAST, no hits are obtained. Indeed, as suspected, although our goal was to perform a covariant mutation that preserves the structure, it can be verified using *mfold* or *RNAfold* [53,56] employing the latest thermodynamic parameters [58] that the whole structure collapses because of energy considerations. This presumably explains the absence of any BLAST hits and also illustrates the importance of structure using a case of the preQ1 riboswitch.

Finally, in a different structure-preserving mutation scenario when again our starting point was the wild-type sequence of the queC-binding aptamer, we obtain the following mutated sequence that is predicted by [53,56] to fold to the same structure of the preQ1 riboswitch:

AAGTGCGATATGCTTGGGCCGTTCTAGCTACACCGGCCATAAAAAAGAAAGGA

For this sequence, when inserting it to BLAST, we peculiarly identify among some distant bacillus types a *Tribolium castaneum* (red flour beetle) hit. Closer inspection of the surrounding of this hit in GenBank, adding more nucleotides upstream and downstream and then performing a folding prediction by energy minimization on a sequence equivalent in size to the initial aptamer sequence and suitably aligned, reveals that the above is a false positive hit. This exemplifies once again the notable importance of structure to the riboswitch aptamer function, and in addition such cases of eukaryotic candidates are of considerable interest in the quest for novel eukaryotic riboswitches.

The designed RNA sequences that comprise the output of RNAfbinv can potentially be used, along with docking considerations, to create synthetic riboswitches that are optimized in some way depending on utility goals. Although the main purpose of our suggested method is to detect novel riboswitches in sequenced organisms, involving docking considerations by simulations with tools such as RNALigand [76] can potentially allow us to computationally design aptamer domains that structurally resemble a natural aptamer (in minimizing the Shapiro coarse-grained distance measure [73] by the use of RNAfbinv [70]) and are favorable in their ligand-binding properties for certain applications.

### Predicting a novel eukaryotic candidate for a purine riboswitch aptamer

**Fig 6:**
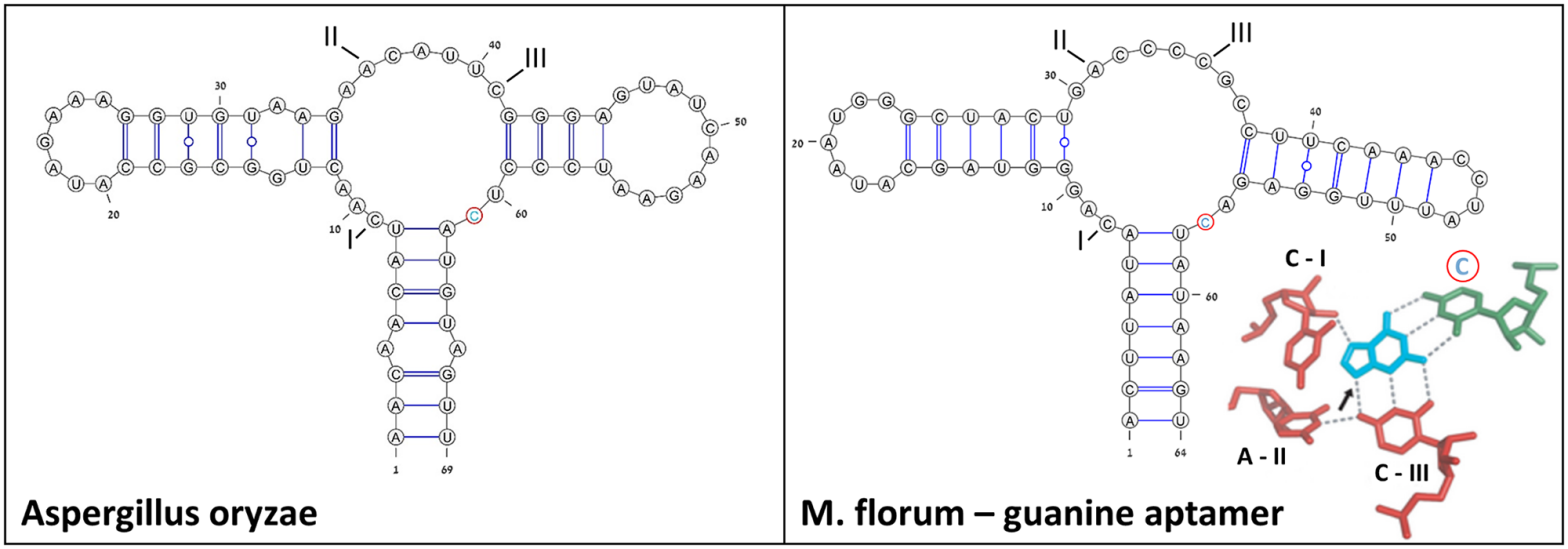
A potential eukaryotic new finding. A potential match found in *Aspergillus oryzae*, in comparison to the *Mesoplasma florum* riboswitch [75]. Essential nucleotides for a potential ligand binding site are marked in both secondary structures. Structure similarity between the two compared structures is relatively high: Base pair distance = 38, Shapiro distance = 4. Sequence similarity is relatively low but the essential nucleotides make it an attractive riboswitch candidate. Similar to [75], a 3D view of the ligand-binding pocket bound to its ligand is depicted on the right-hand side. This predicted purine riboswitch candidate found in fungi is not detected by Infernal.

Additional trials with the general procedure that includes RNAfbinv [70] applied on the purine riboswitch aptamer have yielded a candidate sequence in fungi that cannot be detected by Infernal, or other standard methods tried with for computational riboswitch detection. The new candidate, depicted in Fig 6, is found in *Aspergillus oryzae* and its comparison with the known bacterial *M*. *florum* riboswitch [75] shows interesting prospects. The essential nucleotides for a potential ligand binding site are marked in both secondary structure drawings being compared. Structure similarity between the two compared structures is relatively high while sequence similarity is relatively low, but the essential nucleotides for ligand-binding that are marked in the multi-branch loop make it an attractive riboswitch candidate. As a complementary check, we inserted the new candidate sequence to a webserver called RNAbor that is used for analyzing structural aspects of RNA switches [46] and observed multiple predicted alternative states. The sequence of the known *M*. *florum* riboswitch also possesses more than two alternative states when inserted to RNAbor and therefore the new candidate seems compatible in that regards. Moreover and most significantly, the location of the new candidate sequence in *Aspergillus oryzae* RIB40 in GenBank is near a gene that corresponds to a transporter. This fact is consistent with the observation in [72] that the known *M*. *florum* riboswitch to which our candidate is compared in Fig 6 regulates the COG2252 gene, a class of transporter. Further observations and experimental checks will be carried out and are beyond the scope of the present work that is focused on the computational methodology.

## Conclusion

A new structure-based method for riboswitch detection is presented. The method relies on minimum free energy folding predictions, yet it is highly efficient because the folding predictions are performed only once on the query and thereafter, BLAST searches on computed pre-designed sequences are carried through. The pre-designed sequences are calculated using a flexible inverse RNA folding program that may take seconds/minutes on a standard PC depending on the length of the input structure. An extended inverse RNA folding solver called RNAfbinv [70] is used to perform this task with suitable constraints for the search problem. The method is validated on the purine riboswitch guanine-binding aptamer domain. It is able to detect known purine riboswitches in bacteria, as well as new ones that are not available in Rfam, some of which are potential candidates that are undetected by Infernal and other standard methods for computational riboswitch detection.

Future work is planned to extend this method towards more aptamer domains and other conserved domains in general. Moreover, the importance of applying this approach to riboswitch domains is of high significance at this time especially because of the demand for new approaches that could potentially be able to locate novel eukaryotic riboswitches.
